# Robust autofocusing for scanning electron microscopy based on a dual deep learning network

**DOI:** 10.1038/s41598-021-00412-5

**Published:** 2021-10-22

**Authors:** Woojin Lee, Hyeong Soo Nam, Young Gon Kim, Yong Ju Kim, Jun Hee Lee, Hongki Yoo

**Affiliations:** 1 Department of Mechanical Engineering, KAIST, Daejeon, 34141 Republic of Korea; 2 COXEM Co. Ltd., Daejeon, 34025 Republic of Korea

**Keywords:** Scanning electron microscopy, Software

## Abstract

Scanning electron microscopy (SEM) is a high-resolution imaging technique with subnanometer spatial resolution that is widely used in materials science, basic science, and nanofabrication. However, conducting SEM is rather complex due to the nature of using an electron beam and the many parameters that must be adjusted to acquire high-quality images. Only trained operators can use SEM equipment properly, meaning that the use of SEM is restricted. To broaden the usability of SEM, we propose an autofocus method for a SEM system based on a dual deep learning network, which consists of an autofocusing-evaluation network (AENet) and an autofocusing-control network (ACNet). The AENet was designed to evaluate the quality of given images, with scores ranging from 0 to 9 regardless of the magnification. The ACNet can delicately control the focus of SEM online based on the AENet’s outputs for any lateral sample position and magnification. The results of these dual networks showed successful autofocus performance on three trained samples. Moreover, the robustness of the proposed method was demonstrated by autofocusing on unseen samples. We expect that our autofocusing system will not only contribute to expanding the versatility of SEM but will also be applicable to various microscopes.

## Introduction

Scanning electron microscopy (SEM) produces high-resolution images from secondary electrons generated by projecting focused electron beams on a sample surface^1^. With subnanometer spatial resolution, SEM serves as an important tool to provide structural insight into samples^2^ in fields such as materials science, basic science, and nanofabrication^3^. The quality of the scanned surface image highly depends on SEM parameters, including working distance (WD), magnification, brightness, and contrast. The optimal values of these parameters are also different according to the type of sample being scanned, the height of the sample stage, and the intensity of illumination. In particular, WD, which controls the image focus, has a significant impact on image quality. Therefore, in order to provide optimally focused SEM images with high quality regardless of a user’s level of expertise, a robust autofocus system that can control these parameters is of importance for broadening the variety of research applications.

**Figure 1:**
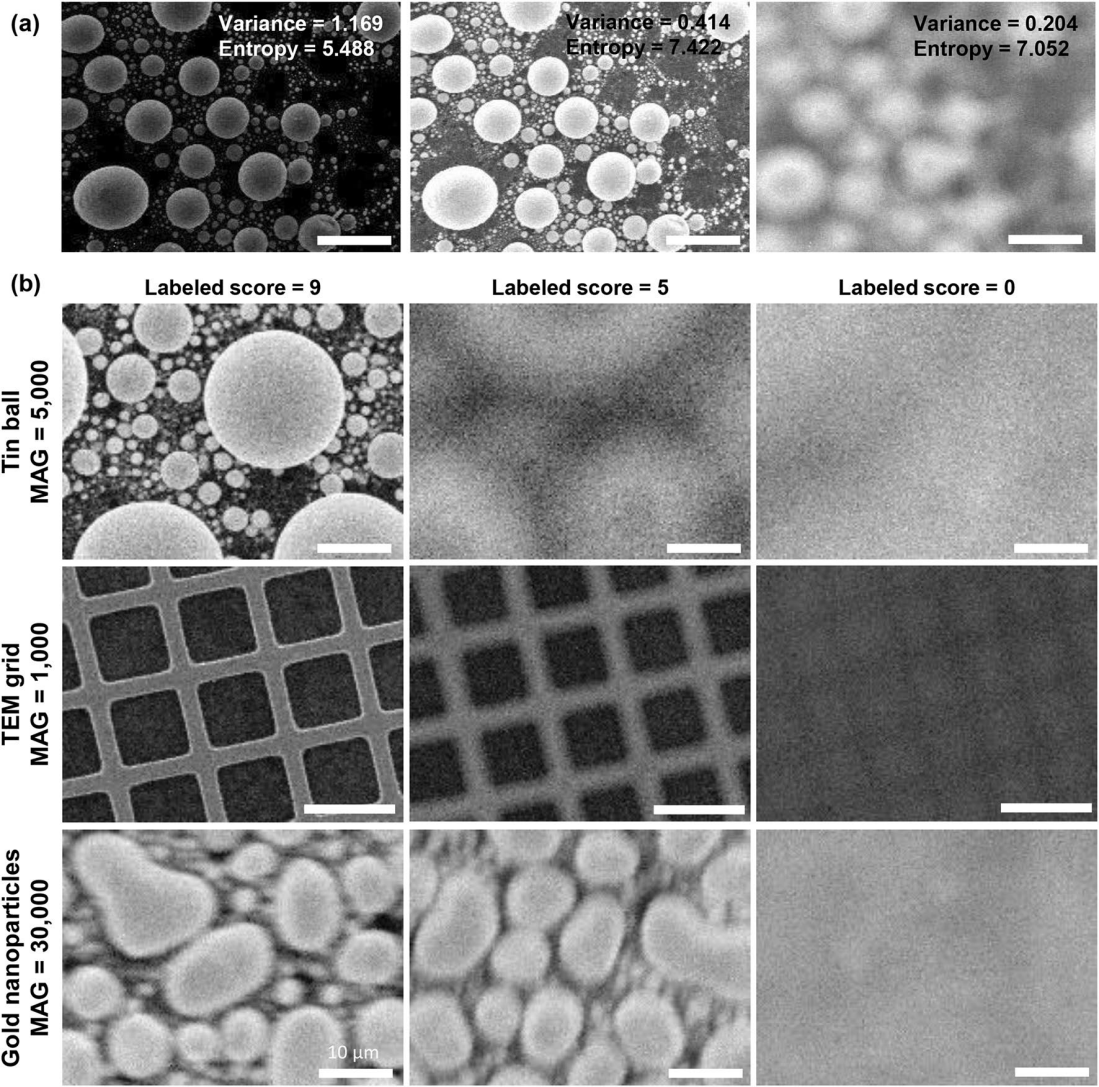
Example SEM images. (**a**) SEM images of tin ball samples with their variance and entropy values. The first two images are the well-focused images but acquired under different brightness and contrast levels, and the third image is the out-of-focus image but acquired under the same brightness and contrast levels as the second image. In general, relatively large variance and entropy means well-focused, but the variance and entropy of the first and second images differ significantly due to differences in brightness and contrast. Therefore, it is difficult to evaluate absolute focus using these values. Also, although the third image is out of focus, the entropy value is higher than first image. (**b**) SEM images from three samples (tin balls, TEM grids, and gold nanoparticles) used in the training. Each column represents images labeled with scores of 9, 5, and 0 from the left, respectively. Because SEM images of gold nanoparticles were scanned at higher magnification, the image scored as 9 inevitably has a lower quality than the other samples. Specifically, the gold nanoparticles image scored as 9 appears similar to the tin balls and TEM grids images with a score of approximately 6. All scale bars except for the gold nanoparticles marked with 10 μm are 100 μm.

Autofocus systems are being actively studied across different fields in which optical microscopes and SEM are used. For example, Geusebroek, et al.^4^ implemented an autofocus system that moves the z-axis stage by a predefined amount in an optical microscope through a focus score defined based on the image gray pixel values and Gaussian derivatives. Sun, et al.^5^ compared the autofocus system that focuses the image based on various image quality metrics. DiMeo, et al.^6^ proposed autofocus control system based on Gaussian shaped focus measure curve and adaptive hill-climbing method. Elsewhere, Harada, et al.^7^ developed a closed-loop autofocus system for SEM through a customized bandpass filter. Moreover, recently, deep learning-based autofocus systems that have shown powerful performance in image processing are being actively studied^8–10^. Luo, et al.^11^ and Na, et al.^12^ respectively proposed an autofocus method for optical microscopy and SEM that receives a defocused image as an input and outputs a virtual focused image based on deep learning. Li, et al.^13^ developed a deep learning model that estimates the appropriate objective lens position by receiving two defocused images for light-sheet fluorescence microscopy. In addition, Jang, et al.^14^ attempted autofocus SEM based on deep reinforcement learning using SEM parameters. However, there are main limitations to the application of the previously studied autofocus systems to SEM. First of all, most autofocus systems have been applied to maximize the sharpness of the image^5,15,16^, using conventional image quality evaluation metrics such as image variance, image entropy, and image gradient^17,18^. Specifically, these image quality metrics are used as evaluation criteria for a focus evaluation system or a single-image super-resolution method^19,20^. These image quality metrics are either defined mathematically using image pixel values or determined by comparison with a reference image. However, there are limitations to its application to autofocus systems. For instance, even when well-focused, widely used evaluation metrics such as the image variance and entropy values highly depend on the brightness or contrast of the image, and these are inappropriate for use as absolute criteria for the autofocus system (Fig. 1a). It is therefore difficult to distinguish between well-focused and out-of-focus cases using these values. Second, even though most conventional autofocus systems have been successfully studied and applied to optical microscopes, SEM is difficult to apply because image quality is more sensitive to parameter changes due to its inherent high magnification. In general, when comparing an image sharpness metric between images from different focal lengths, the focal range that maintains the sharpness metric is relatively wider in an optical microscope than in SEM^21,22^. In addition, SEM is based on electron beams instead of light, making it much more vulnerable to noise than optical microscopes due to factors such as electron absorption properties and charge-damaged artifacts^23,24^. Furthermore, deep learning-based autofocus research is also difficult to be widely applied to SEM, because most of the studies have been applied to optical microscopes based on numerical image quality metric, which have the aforementioned limitations^11,13^. Even when applied to SEM, there are limitations in the range of available WD and magnification^12,14^. Therefore, a new autofocus system tailored to the technical characteristics of SEM is required.

In this study, we propose an autofocus method for a SEM system based on a dual deep learning network consisting of an autofocusing-evaluation network (AENet) and an autofocusing-control network (ACNet). The AENet evaluates the focusing quality of SEM images according to WD in various environments and provides an absolute criterion for the autofocus system, and ACNet delicately controls the SEM parameters online at all magnifications and lateral positions within the recognizable range of the sample. Since SEM is very sensitive to parameter changes compared with other optical microscopy techniques, precise control and immediate feedback are important. In conjunction with the AENet, the ACNet allows automatic delicate control of the SEM parameters by inputting the AENet results, which overcomes the limitation of the traditional image quality evaluation. The results of this study are expected to be applicable not only to SEM but also to optical microscopy and other microscopy fields in that the dual deep learning network is combined to replace traditional quality metrics and directly control the system variables.

## Results

**Figure 2:**
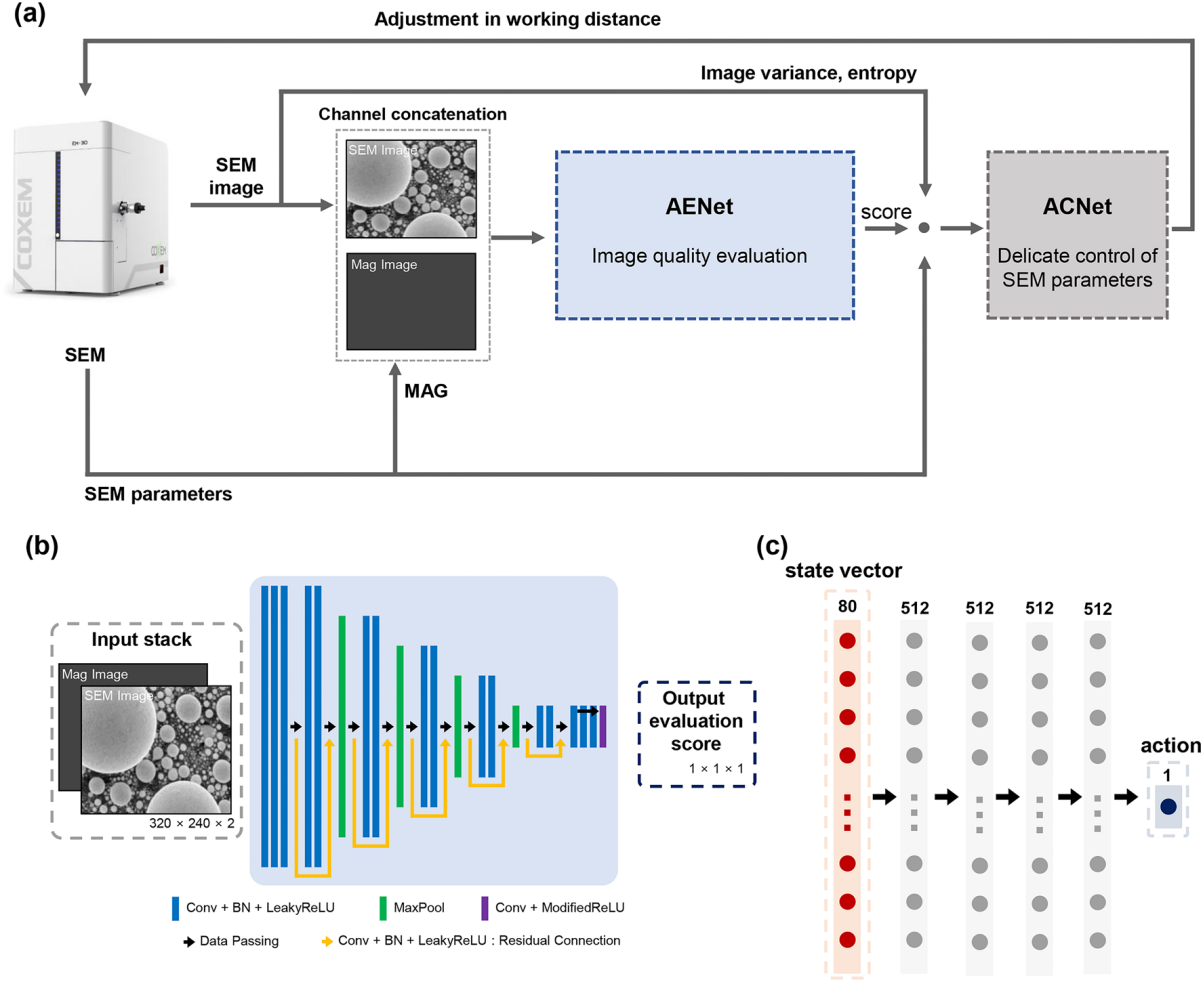
(**a**) Overall process and architectures of the proposed autofocus SEM based on a dual deep learning network. The SEM image and the corresponding SEM parameters are transferred to the dual network. The autofocusing-evaluation network (AENet) evaluates image quality according to the given working distance based on the current image and the mag-image that is filled with the normalized magnification value of the same size as the current image. The autofocusing-control network (ACNet) determines the proper action, which means how much the working distance will be adjusted using a combination of AENet score, SEM parameters (working distance (WD) and magnification (MAG)) and traditional image quality metrics (image variance and entropy). Then, the amount of working distance to be adjusted is transmitted back to the SEM via a socket connection. (**b**) Schematic of the proposed AENet architecture. The score is determined from the concatenation of the given image and its mag-image. The AENet consists of 21 layers (consisting of 17 convolution layers and four max-pooling layers) and five residual connection layers. Each convolution block consists of a convolution layer, 2D batch normalization, and leaky rectified linear units (Leaky-ReLU) activation function. (**c**) Schematic of the proposed ACNet architecture. ACNet determines how much to adjust the working distance by inputting the combination of the AENet score and the aforementioned parameters of the given image. ACNet comprises five fully connected layers, and each layer has 1D batch normalization and Leaky-ReLU activation function.

The overall process of the proposed autofocus method based on the dual deep learning is presented in Fig. 2a. First, the acquired SEM image and the corresponding parameters, including magnification and working distance (WD), are transferred through a USB interface using SEM control software. The AENet provides a focusing quality score of the image by taking the SEM image and the mag-image that is generated by filling with the normalized magnification value as input. Then, the ACNet determines how much the WD needs to be adjusted at the current state using a combination of the AENet score, the SEM parameters, and additional image quality metrics. The result of the ACNet is transmitted back to the SEM via a socket connection to control the WD.

Training datasets were obtained for three different samples (tin balls, transmission electron microscopy (TEM) grids, and gold nanoparticles) using a commercial SEM system (EM-30, COXEM, Daejeon, Republic of Korea) (Fig. 1b). The datasets for each model were collected from two different settings: Dataset 1 and Dataset 2. The Dataset 1 was acquired to train the AENet. To provide ground truth when training the AENet, each image was labeled with a score between 0 and 9 by SEM experts. A perfectly focused image was assigned a score of 9, and fully out-of-focus image scored 0. The Dataset 2 for the ACNet training was additionally obtained by varying the WD for the fixed lateral position and magnification to embody image quality variations with respect to the change in WD. The AENet is based on convolutional layers consisting of a total of 21 layers (Fig. 2b), while the ACNet is based on five fully connected layers (Fig. 2c). Implementation details can be found in the “Method” section. The performance of each network was tested offline. Then, we combined the networks and applied them to the online SEM environment to demonstrate autofocusing capabilities.

### Offline performance test

#### Autofocusing-evaluation network (AENet)

To overcome the limitations of existing metrics and to assess image focusing quality independent of magnification, we introduced the AENet, which enabled deep learning–based evaluation of image focusing quality with a score awarded from 0 (out of focus) to 9 (in focus) for a given SEM image. In general, magnification directly affects the image quality. For example, at a high magnification of 20,000, the best image inevitably has a lower quality than the best image at a low magnification of 5000 because images from the higher magnification are more susceptible to noise and the physical resolution of the SEM equipment is limited. The AENet therefore received both an image and a magnification as an input to reflect the difference in image quality according to the magnification by concatenating an image and the corresponding mag-image in the channel dimension.

**Figure 3:**
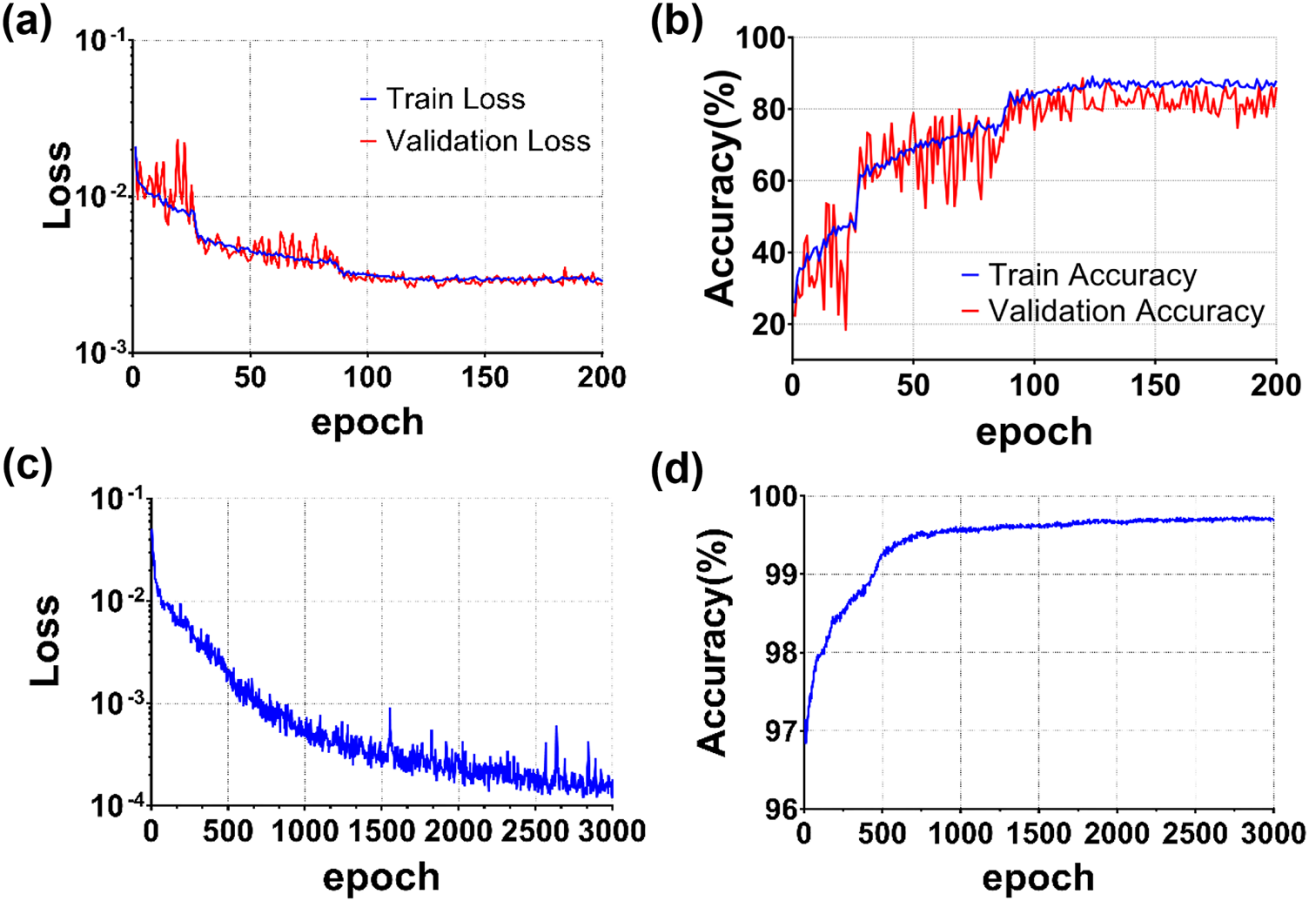
(**a**, **b**) Represent plots of training loss and accuracy of the proposed AENet in the training and validation for each epoch, respectively. Training loss was calculated using mean square error (MSE) between the score given by experts and the score given by AENet, and accuracy was measured based on the output being correct in the case that it was within ± 1 of the label. (**c**, **d**) represent plots of training loss and accuracy of the proposed ACNet in the training, respectively. There was no validation set because random noise was added to the data for each epoch. Training loss was calculated using MSE between the target action and action selected by ACNet, and accuracy was measured based on the selected action being correct in the case it was within ± 5 of the target action.

As shown in the loss and accuracy curves in the training and validation sets for the AENet (Fig. 3a), the AENet converged successfully in training without overfitting, and the training and validation accuracy increased and reached plateaus. Here, the accuracy of the AENet was evaluated to be correct if it was within the range of 1 based on the ground truth assigned by the SEM experts, and the degree of difference was not considered. The final accuracy was around 80%, when the training converged (Fig. 3b). The relatively low final accuracy could be attributed to the fact that the data poorly labelled by SEM experts might be included in the training dataset.

**Figure 4:**
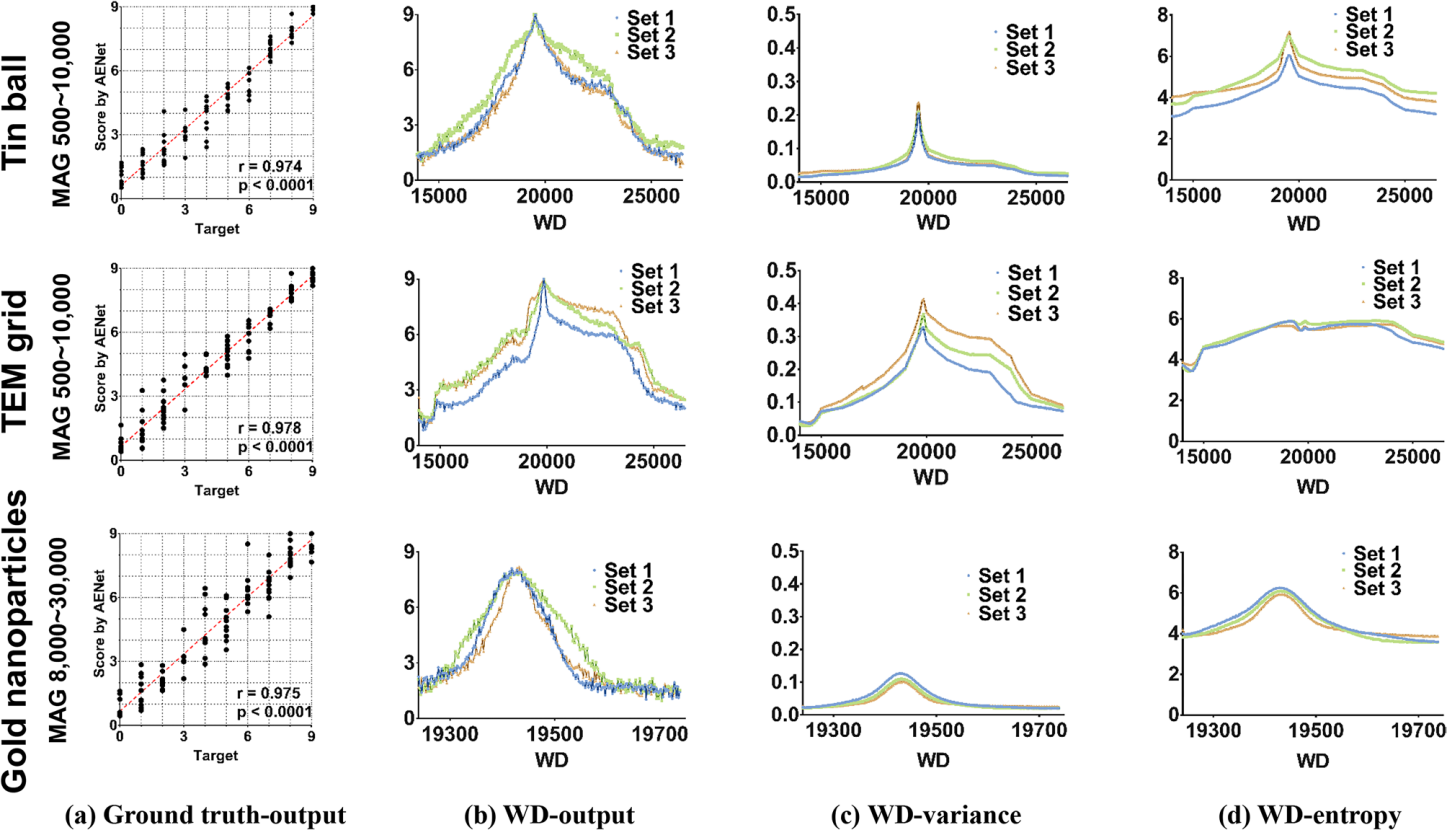
Focus evaluation by the trained AENet. (**a**) Correspondence plots of the results for each sample evaluated by the trained AENet. The x-axis denotes the score evaluated by the SEM experts, and the y-axis denotes the resultant scores of AENet’s evaluation. The result shows a good linear tendency between the experts’ score and the estimated label. The linear fitted lines (red dashed line) also indicate that the results are highly correlated. (**b–d**) Plots of the quality evaluation results according to the working distance (WD). The x-axis denotes WD. (**b**) WD vs. AENet score, (**c**) WD vs. variance, (**d**) WD vs. entropy.

The performance evaluation results for each sample are shown in Fig. 4a. The mag-image that was concatenated with the SEM image would benefit the absolute evaluation of focusing quality regardless of the magnification so that perfectly focused SEM images obtained at various magnifications could be evaluated as close to the score of 9 as possible (Fig. 4a). The scores predicted by the AENet were highly correlated with those labeled by the SEM experts, with correlation coefficients of 0.974, 0.978, and 0.975 for tin balls, TEM grids, and gold nanoparticles, respectively. We further evaluated the AENet by measuring mean square error (MSE), accuracy, and precision for each sample, including tin balls, TEM grids, and gold nanoparticles. When calculating the accuracy, as in the training phase, the AENet output was regarded as a correct answer if it was within ± 1.0 of the ground truth assigned by the SEM experts. Precision was defined as how different the score (network output) is from the label expressed as follows:

1 $$ {\text{precision}} = \sum\limits_{i} {(1 - \left| {\widehat{{y_{i} }} - y_{i} } \right|/10)}  $$

where $\widehat{{y}_{i}}$ and ${y}_{i}$ are the predicted score and the labeled score, respectively. The performance evaluation results are summarized in Table 1. Despite the good correspondence, however, MSE and accuracy measured in the gold nanoparticles were worse than those in the other two samples (Table 1). We speculated that there were two reasons for the performance degradation in gold nanoparticles: First, the aforementioned labeling discrepancy in the gold nanoparticles was more prominent even in high scores. Second, the images with gold nanoparticles were obtained at much higher magnification where noise is more likely to affect image quality. Nevertheless, the precision assessing the degree of deviation between the label and the predicted scores was calculated as quite high in all three samples (Table 1), suggesting that the readouts from the AENet could be sufficiently used as the absolute SEM image evaluation metric, which is robust to the variation of the magnification and samples.

**Table 1:** Offline test results of autofocusing-evaluation network (AENet) and autofocusing-control network (ACNet).

	Tin ball	TEM grid	Gold nanoparticles
**AENet result**			
MSE	0.047 ± 0.017	0.045 ± 0.021	0.071 ± 0.032
Accuracy	0.920 ± 0.428	0.880 ± 0.423	0.731 ± 0.325
Precision	0.943 ± 0.351	0.944 ± 0.452	0.931 ± 0.423
**ACNet result**			
Average of selected score	8.468 ± 1.015	8.605 ± 0.899	7.788 ± 1.380
Average of score diff	0.158 ± 0.414	0.152 ± 0.310	0.068 ± 0.071
Average of iteration	9.100 ± 4.154	9.100 ± 4.128	9.947 ± 4.075
MSE	0.020 ± 0.008	0.021 ± 0.012	0.001 ± 0.000

Figures 4b–d show the evaluation results of SEM images in Dataset 2, which were obtained by varying the WD at a randomly selected magnification and lateral position of the stage. As the WD increases, the AENet provides the absolute evaluation on the SEM image focusing quality by the 0–9 scale, and clear peaks are observed around the optimal WD for each sample (Fig. 4b). Comparing the evaluation by the AENet score with the image variance and entropy (Figs. 4c,d), although both metrics also exhibited a similar tendency along with the WD showing their maximum around the optimal WD, their maximum values were different depending on the magnification and sample being scanned, revealing that the image variance and entropy were not suitable for use as the absolute evaluation indicators. This is an important point, since this study aimed to develop robust autofocus SEM for various samples through a single system.

#### Autofocusing-control network (ACNet)

The ACNet directly controls the WD of the SEM using the results of the AENet. Specifically, the ACNet produces an amount of WD to be adjusted to approach the best focusing based on the present and past values of the AENet score, WD, image variance, and image entropy. We defined the amount of WD that the ACNet produces as action of the ACNet. The input of the ACNet was defined as state, as it represents the current and the past SEM environment. Each state comprised a combination of the aforementioned parameters and their differences with respect to those of the previous state, since we postulated that the best focus can be estimated using the current and past information. Since the SEM image quality from the higher magnification becomes highly sensitive even to small changes in the WD, the maximum action size determined by the ACNet was adaptively adjusted according to the given magnification for fine-tuning of WD. The ACNet was first trained and tested in an offline manner using Dataset 2 with pre-acquired SEM images and the corresponding parameters, and then the trained ACNet was applied to an online environment to verify whether it can achieve the autofocusing in the online SEM.

As shown in the loss and accuracy curves in the training sets (Fig. 3c,d), the ACNet converged successfully in training. Here, the accuracy of the ACNet was evaluated to be correct if it was within the range of 5 based on the target action, and the degree of difference was not considered. As a result of the training, the trained ACNet was able to successfully determine how much the current WD should be adjusted to find the best in-focus image under conditions of random magnification and lateral position of the stage for the three different samples. Table 1 summarizes the results of the offline performance test for the ACNet. As shown in the table, the averaged final score provided by the ACNet was evaluated to be above 8. In the case of gold nanoparticles, the average score value was slightly lower than that for the other samples because the maximum score itself was inherently slightly low due to the noise susceptibility and the limited resolution at high magnification. However, the difference between the final selected score and the actual best score was also measured as very low, and the MSE between the output and the target action was measured as low for all three samples. In addition, the average number of iterations required to achieve the autofocusing was only about nine, which means the ACNet could find the best in-focus image very quickly. We further investigated whether both AENet score and conventional image quality metric contribute to the improvement of the autofocusing capability. As summarized in Supplementary Table S1, the proposed ACNet achieves superior performance compared with the ACNet trained without either the AENet score or the conventional image quality metric, in terms of all the evaluation metrics, revealing that the AENet score and the metric can serve as an absolute criterion and a complementary indicator to describe the image quality for the autofocusing, respectively. Intriguingly, the average number of iterations required to achieve the autofocusing was noticeably decreased in the proposed ACNet. Taken together, these results suggest that the proposed ACNet can determine the proper action under various conditions and samples by recognizing the score change tendency according to the WD.

### Online performance test

**Figure 5:**
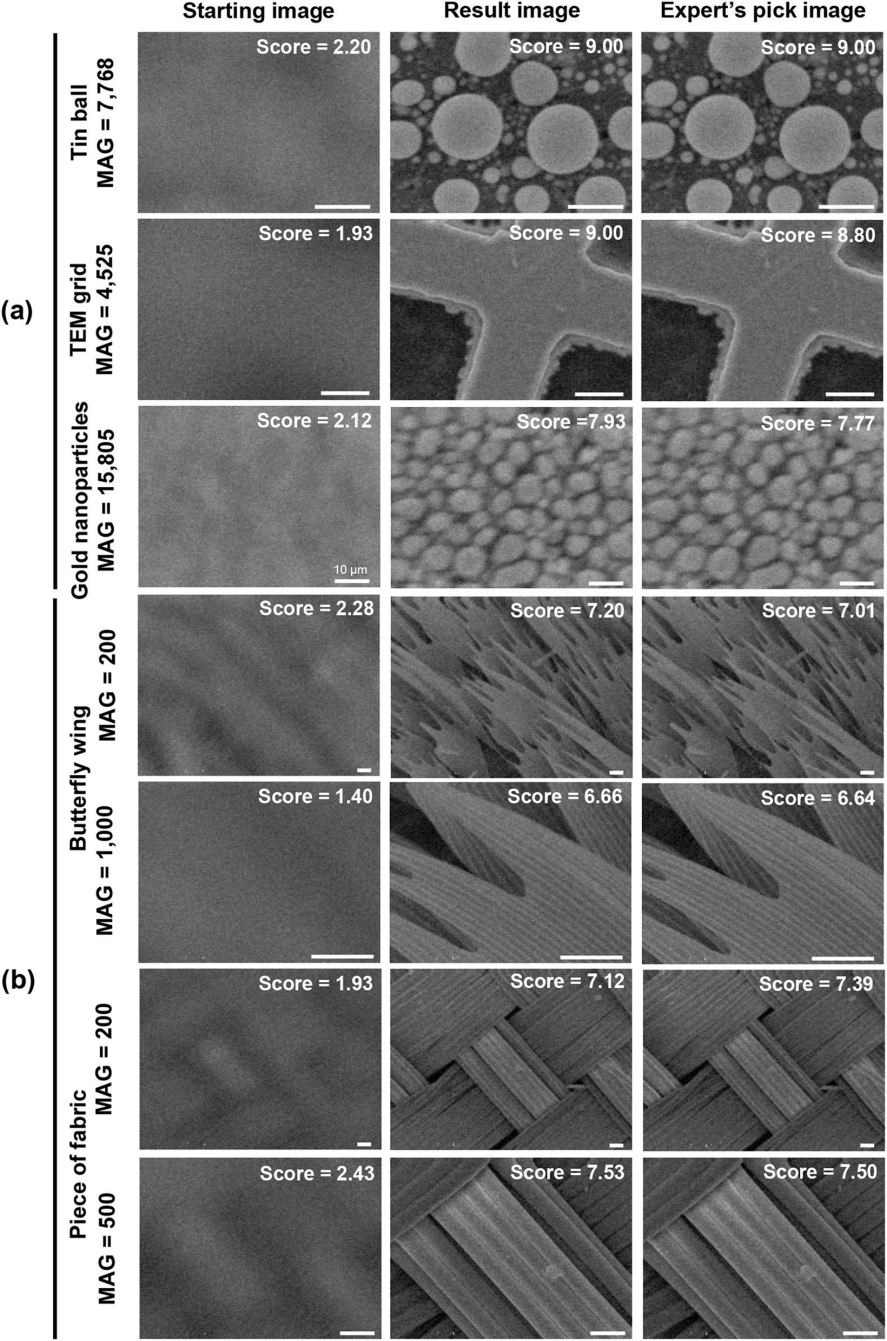
(**a**) For visual comparison, the starting images (left column), the images provided by the autofocus SEM (center column), and those manually selected by SEM experts (right column) are shown. (**b**) Examples of autofocus SEM result for the untrained samples. The upper two rows show a butterfly wing; the bottom two rows show a fabric piece. Each sample was scanned at a magnification similar to that of the TEM grid and tin ball. All scale bars except for the gold nanoparticles marked with 10 μm are 100 μm.

The autofocus SEM online performance was evaluated by applying it to the actual SEM system to demonstrate whether the proposed dual network could find a perfectly in-focus image in various environments. The online performance test was conducted 100 times on each sample under a randomly selected lateral position and magnification. Note that even with the same sample, if the lateral position of the stage changed, detailed structural information of the scanned image changed. The results of the performance test are summarized in Table 2. The average of the final selected scores was also high, comparable with that in the offline test for all samples (Table 1). Even though the result of the gold nanoparticles at high magnification was lower than that of other samples, the best in-focus image could be obtained by the autofocus SEM (Fig. 5a). The average iteration number was also similar to that in the offline test, but its standard deviation was somewhat higher. We speculate that this was due to an unexpected images from the edge and the dust particles of the sample being scanned in the process of randomly positioning the sample. The images provided by the autofocus SEM system and those manually selected by the SEM experts were compared in Fig. 5a. The result clearly supported that the autofocusing dual network could be operated in a robust manner in that there was no significant difference in the AENet scores between the two images and that it could provide images even with superior quality compared with those selected by the users (Table 3).

**Table 2:** Autofocus online performance test results with AENet and ACNet conbined.

	Tin ball	TEM grid	Gold nanoparticles
Average of selected score	8.534 ± 1.071	8.568 ± 0.564	7.458 ± 1.002
Average of iteration	10.743 ± 6.128	13.300 ± 5.424	10.574 ± 3.511

**Table 3:** Comparison of online performance test for the trained samples and unseen samples. The images provided by the autofocus SEM and those manually selected by the SEM experts were evaluated by the AENet for each sample.

	Trained sample			Unseen sample	
	Tin ball	TEM Grid	Gold nanoparticles	Butterfly wing	Piece of fabric
Average score of images provided by autofocus SEM	8.814 ± 0.255	8.480 ± 0.456	7.977 ± 0.999	6.528 ± 0.632	6.406 ± 0.695
Average score of images provided by SEM experts	8.773 ± 0.312	8.520 ± 0.431	7.250 ± 0.495	7.314 ± 0.789	7.440 ± 0.622

To investigate whether the autofocus SEM could be applied to a wide variety of samples, autofocusing was further performed on unseen samples in the training phase. A butterfly wing and a piece of fabric with completely different structural information to the three samples used in the traing were employed and scanned at similar magnifications to the tin balls and TEM grids. The images provided by the autofocus SEM method for these two samples and those selected by SEM experts were compared in Fig. 5b. Consistent with the result in Fig. 5a, the dual network could find images with similar quality to those identified by the SEM experts (Table 3), implying the combination of AENet and ACNet could successfully find the well-focused WD for the unseen samples. The autofocusing SEM demonstration videos for all presented samples are available in Supplementary Movies S1–8.

## Discussion

In this study, we proposed a robust online autofocus method for SEM based on a dual deep learning network comprising the AENet and the ACNet. The AENet was able to evaluate the focusing quality of the image regardless of the magnification using the concatenation input of the SEM image and the corresponding mag-image, which cannot be provided by conventional image evaluation metrics. The ACNet was able to directly control the SEM in real time based on the AENet outputs. The quantitative results of the dual network–based autofocusing on both offline and online environments showed that the combination of the AENet and the ACNet could successfully find the best in-focus images; the results were comparable with, or even better than, the selections by SEM experts. The proposed autofocusing method was also validated on two additional unseen samples that were not used for training, ensuring its robustness to various samples.

The critical role of the AENet in the autofocus system is to provide an absolute criterion based on the degree of focus of the scanned SEM image. In other words, the autofocusing performance was highly dependent on the outputs of the AENet. Although the AENet was demonstrated to successfully evaluate the image in this study, the limited dataset only comprised three representative samples, and inconsistent labeling could limit the performance of the AENet. Note that constructing the dataset by acquisition and annotation of images involves labor-intensive and time-consuming manual efforts, which can potentially cause reduced reliability and reproducibility. Since discrepancy that can occur in a manual labeling process by readers has been problematic in other deep learning applications, several studies have been conducted to efficiently train from noisy labels^25–28^. Once labeling inconsistency is reduced by re-labeling, we expect the performance of the AENet will be improved. Furthermore, it will also be possible to re-train the trained AENet to work better on various samples using newly acquired sample images^29–31^. In the near future, we can expect that constructing a new dataset for the re-training will be facilitated by storing image data at online databases by end-users, and labeling images semi-automatically using the pre-trained AENet network.

For the ACNet, we postulated that finding the optimal WD is a problem with a correct answer (ground truth), which can be solved using a supervised learning–based deep learning model. Therefore, the ACNet was trained using the mean square error loss calculated by comparing the output and the target action. Therefore, the ACNet was designed to solve a relatively low-dimensional problem with a clear ground truth using the mean square error loss calculated by comparing the output and the target action. However, the proposed autofocus SEM method has an inherent limitation that real-time active learning based on given environments is infeasible, and thus re-training may be required to achieve comparable performance on a variety of samples. In general, deep reinforcement learning, which provides the unknown optimal actions by interacting various environments with multiple dimensions, is widely used in control fields, such as in robotics control^32–34^. State-of-the-art reinforcement learning methods, such as deep deterministic policy gradient^35^ and proximal policy optimization algorithms^36^, have been extensively studied in various fields^37^. We expect that applying the reinforcement learning strategies, which determine the optimal action using the policy function actively learned from a given environment without ground truth will be an alternative for real-time updating to enhance robustness to unseen samples.

While SEM allows for nanoscale investigation of samples, the acquisition of high-quality images is difficult to achieve by general users due to a lack of expertise in SEM. Since the autofocus method for SEM proposed in this study can fully replace the need for expert-level skills, it could make a valuable contribution to broadening the versatility of SEM in the future. We expect that our method can be applied not only to SEM, but also to other optical equipment or microscopy fields if trained on other samples in different environments and conditions.

## Methods

### Datasets

To prepare datasets for the training, each sample was obtained to be evenly distributed within a predetermined magnification range by randomly translating the lateral position. The images of the tin ball and the transmission electron microscopy (TEM) grid were acquired at magnifications between 500 and 10,000. Since individual gold nanoparticles could be identified at higher magnification, the images were acquired at increased magnifications between 8,000 and 30,000.

When acquiring Dataset 1, the lateral position and the magnification were randomly selected in the predetermined ranges for all of the images. The total number of obtained images in Dataset 1 with a size of 640 × 480 pixels was 30,000 with 10,000 for each sample. Each image was scored between 0 (fully out-of-focused) and 9 (perfectly focused) by the SEM experts, which served as the ground truth, an example of which is shown in Fig. 1b. A total of 30,000 Dataset 1 was randomly divided into 70% data for the training set and 15% each for the validation set and the test set.

To enable fast feedback when autofocusing, the SEM machine used in this study could increase the frame rate by sacrificing the image quality and reducing the image size of 320 × 240 pixels. Note that to reflect this in the dataset, SEM images in Dataset 2 were obtained in the faster imaging mode. Dataset 2, consisting of 91 sets with 250 images each, was not divided into training and validation test sets because Dataset 2 added a different random noise at each epoch, giving it unseen data for each epoch. The other conditions were the same as those for Dataset 1.

### Autofocusing-evaluation network (AENet)

#### Preprocessing

Prior to the training and validation procedures, median filtering was preprocessed with a 3 × 3 sized kernel. To improve learning efficiency from the viewpoint of a gradient descent algorithm and to eliminate image variations^38^, each image was normalized with its mean value and standard deviation as follows:

2 $${I}^{{\prime}}=\frac{I-\mu }{\sigma }$$

where $I$ and ${I}^{{{\prime}}}$ are the original and normalized images, respectively, and $\mu$ and $\sigma$ are mean and standard deviation of the original image, respectively. After the preprocessing, each image was randomly cropped to 320 × 240 pixels and flipped in either the vertical or horizontal direction to augment the training dataset.

#### Training strategy

The architecture of the AENet was designed with a customized structure by adopting the residual connection in the ResNet architecture^39^, consisting of a total of 21 layers with 17 convolutional layers and four max-pooling layers and five residual connections. The schematic of the model is shown in Fig. 2b, and the details of the AENet architecture are presented in Supplementary Table S2. Each layer employed the leaky rectified linear units (Leaky-ReLU) as an activation function, and 2D batch normalization was applied to accelerate and stabilize training. As the last activation function, modified ReLU activation, in which the activation values larger than nine were thresholded to nine, was applied. Since the AENet was fully convolutional and thus the output size was directly determined by the input size, in the case of taking an input whose size was larger than 320 × 240 pixels, the final evaluation score was selected as a median value of the AENet output. We used the mean square error (MSE) loss function for training the AENet so that the parameter weights of the network converged to the optimal value during training. Accuracy was monitored during the training to see whether overfitting was present. Accuracy was measured the same as in the evaluation process. Initiating with a batch size of 90 and an initial learning rate of 0.01, which was decayed with the factor of 0.1 if there was no further decrease in the loss in the validation set for the last 10 epochs, we trained the network for 200 epochs with no early stopping.

### Autofocusing-control network (ACNet)

#### Data for ACNet

To describe the state of the current SEM environment, an evaluation score provided by the AENet, image variance, and entropy were combined as the input of the ACNet. Here, image variance and entropy were defined as follows:

3 $$\mathrm{variance}\left(\mathrm{I}\right)= \frac{\sigma (I)}{\mu (I)}$$

4 $$ {\text{entropy}}\left( {\text{I}} \right) =  - \sum\limits_{i}^{{n - 1}} {p_{i} } \log _{2} p_{i}  $$

where $I$ is a given SEM image, and $\sigma$, $\mu$, and ${p}_{i}$ are the standard deviation, mean operator, and normalized histogram frequency, respectively. Supplementary Figures S1a–c show an example of ACNet data. The evaluation scores, including AENet score, image variance, and entropy, vary according to the WD. The evaluation scores have the highest value at the best focus and gradually decrease as the WD moves away from the best focus. To effectively train the ACNet in an offline environment, state should be defined over a continuous range of WD. However, the offline dataset, i.e. Dataset 2, was established from sparse sampling within a specific range of WD (Supplementary Fig. S1a). In order to provide a continuous state along the WD, the values of AENet score, image variance, and entropy from each set were filled with spline interpolation (Supplementary Fig. S1b). While SEM images are highly susceptible to noise and AENet score could be affected by noise, the spline interpolation naturally reduces the noise in the dataset. To reflect the noise characteristics and to make the offline environment more realistic, random noise was added to each interpolated dataset (Supplementary Fig. S1c).

#### Training strategy

The ACNet determines how much the current WD should be adjusted to achieve the best focusing, which is defined as action here. In this process, the inherent property of SEM extremely limits the depth of focus compared with optical microscopy, and there are considerable artifacts in that the image continuously shakes due to its electron-based characteristics^23^. In addition, the maximum action size should be adaptively adjusted according to the magnification because even subtle changes in WD at high magnification could lead to profound differences in image quality. The ACNet was designed as a lightweight architecture with five fully connected layers where 1D batch normalization was applied for the adjustment and the Leaky-ReLU was used as an activation function. The schematic of the ACNet model architecture is shown in Fig. 2c. The state, which is the ACNet input, can be expressed as a vector comprising WD, AENet score, image variance, entropy, and magnification and their differences with respect to the last 20 states. We empirically found that the information of the 20 previous frames is useful for deciding the next action. The last 20 states were initialized with the values at starting WD, and updated in chronological order as it was iterated. In particular, the current value of the WD was not used as state to prevent the ACNet from training a specific fixed WD as the best focal position, we used only the differences of working distances, excluding the current absolute value, as the optimal working distance was almost the same according to the z-axis position of the sample stage in the acquired dataset. Although autofocusing mostly ends within 20 frames, we considered the need for additional exploration due to unforeseen situations. Supplementary Table S3 summarizes all elements of each input state vector. The state can be expressed as a vector of size 1 × 80. Each element of the input state vector was normalized to a normalization factor according to the comprised component to be within the range [− 1,1] for efficient and stable training. The target action, which is the ground truth in the training phase, was defined as the difference between the current WD and the best WD, where the highest AENet score was achieved in the given set. For the efficient adjustment of WD for the best focusing, it was important that the ACNet (1) should find the best WD with as few iterations as possible and that its output (2) should not lead to oscillation around the best WD. Thus, in the case where the target action was considered too large, it was thresholded to the predetermined maximum action size so that too large adjustment on WD could not be made. We found the maximum allowable change in WD that satisfies the aforementioned two conditions was 1200 at the lower magnification. To determine how much the maximum allowable change (or maximum action size) should be reduced at the high magnification, full-width at half-maximum (FWHM) of the measured AENet score profile with respect to WD was calculated from each training set in Dataset 2, which was obtained at various magnifications, as shown in Supplementary Fig. 1d. The calculated FWHM values according to the magnification were fitted to the hyperbolic tangent function expressed as follows:

5 $${{{A}}}_{{{m}}{{a}}{{x}}}\left({{m}}\right)=1200\times \left(-0.485\times {\rm {tan}} h\left(\frac{{{m}}-9000}{1000}\right)+0.515\right)$$

where ${\mathrm{A}}_{max}$ is the maximum action size in WD on the given magnification $m$. We found the maximum allowable change at the higher magnification was expected to be 36. By adaptively selecting the maximum action size according to magnification using the fitted function, robust autofocusing covering a wide range of magnification would be achieved. We imposed the initial searching and terminal conditions for the adjustment of WD by the ACNet to guarantee fast and robust autofocusing. The initial searching process starts at a randomly selected WD, and adjusts the WD with a sufficient interval to find an appropriate initial WD at which the sample under imaging gets identified with the AENet score greater than 2. Then, the ACNet starts to find the optimal, best-focused WD. By the end of the training phase, even if the ACNet succeeded in finding the optimal WD with the AENet score of 9, the training procedure continued for 20 iterations. This helped the ACNet to learn to stay in the high score without oscillation. On the other hand, when the test in the online environment was applied to actual SEM, the condition was changed to stop immediately when reaching a score of 9 and to stop when the obtained score was not updated for five iterations so as to minimize the iteration to find the best focus. The maximum number of iterations was 20. If there was an unexpected object, such as dust on the sample, or an untrained situation was encountered, a countermeasure was prepared so that the autofocusing could be attempted once again. When the final AENet score was less than 3 after finishing the autofocusing, it was considered to be a failure case. We used the MSE loss function so that the parameter weights of the network converged to the optimal value during training. Accuracy was monitored during the training to see whether overfitting was present. When calculating the accuracy, the action was regarded as correct if it was within ± 5 of the given target WD. For efficient training we set aside a memory that stores a history of pairs of past states and the corresponding actions, and for each training epoch, a batch was fetched from memory as much as a predetermined batch size. The learning was started with a batch size of 90 and a learning rate of 0.01 for 3000 epochs with no early stopping.

### Offline performance test for each model

The performance of each network should be evaluated separately before a full validation of the autofocus SEM system with the dual deep learning network. The performance of the AENet was measured using the MSE, accuracy, and precision between the predicted scores and the ground truths. Accuracy and precision were measured the same as in the evaluation process. Through this result, we can demonstrate that the AENet can evaluate the data as having a similar score to the label according to the focus. In addition, linear regression between the target score and the network output score was used. The AENet was also applied to Dataset 2 to assess the image evaluation performance according to the WD. The performance of the ACNet was verified by measuring the MSE between the target action (ground truth) and the action as an output of the ACNet. In addition, the ACNet trained in an offline environment where the optimal WD with the best score was known was validated by comparing the final selected score with the actual best score.

### Online performance test (online SEM connection)

As shown in the overall schematic in Fig. 2a, both trained networks were combined and then applied to the online SEM environment. To justify the performance of the autofocus SEM method, it is important to find a well-focused high-quality image accurately, but the number of iterations required should also be taken into account. Therefore, not only image scores from the final decision but also the number of iterations, that is, the number of frames were used as the metrics for the online performance test. Note that the systemic factors of the SEM itself inevitably cause a time delay in the communication process between the dual network and the SEM machine; the elapsed time was not considered as a metric for performance evaluation. In the online test, since a clear ground truth image of the best focus was not known (unlike the performance test in the offline environment), images autofocused by the dual network were compared with the best in-focus images selected by the SEM experts blinded to the autofocus results. An additional point to consider was that autofocusing based on the dual deep learning network starts after the initial searching. Since the WD range where the sample can be identified was very narrow compared with the actual adjustable WD range in which the SEM can operate, it was important to set a proper starting position for the fast and robust autofocusing. The stride size of initial searching was determined according to the magnification: The stride size at lower magnification was 400, and the minimum size was 12 at higher magnification.

To evaluate the performance of the autofocus SEM for unseen samples in the training phase, we utilized two additional samples: a butterfly wing and a piece of fabric. Although the two samples were scanned at a similar magnification to the tin ball and the TEM grid, they had completely different structural information to demonstrate the robustness of the autofocusing to other samples.

### System implementation

Each network was sequentially optimized using the Adam optimizer with an initial learning rate of 0.01^40^. All of the training methods were implemented using PyTorch (https://pytorch.org/) on a GPU server with four NVIDIA RTX 2080 Ti cards and CUDA V11.1. Using these hardware specifications, the total training time took approximately 2 days for the AENet of 200 epochs and the ACNet of 3000 epochs.

## Supplementary Information

Supplementary Video 1.

Supplementary Video 2.

Supplementary Video 3.

Supplementary Video 4.

Supplementary Video 5.

Supplementary Video 6.

Supplementary Video 7.

Supplementary Video 8.

Supplementary Information 1.
