# Supplementary material for: Robust autofocusing for scanning electron microscopy based on a dual deep learning network
Source: Sci Rep. 2021 Oct 22;11:20933. doi: 10.1038/s41598-021-00412-5 (PMC8536763; doi:10.1038/s41598-021-00412-5)
Supplement: Supplementary file 9 — Supplementary Information 1. [file 41598_2021_412_MOESM9_ESM.docx]

**Supplementary Information**

**Title:** Robust autofocusing for scanning electron microscopy based on a dual deep learning network

A**uthors**: Woojin Lee^1^, Hyeong Soo Nam^1^, Young Gon Kim^2^, Yong Ju Kim^2^, Jun Hee Lee^2^, and Hongki Yoo^1, *^

**Affiliations:**

^1^Department of Mechanical Engineering, KAIST, Daejeon, 34141, Republic of Korea

^2^COXEM Co. Ltd., Daejeon, 34025, Republic of Korea


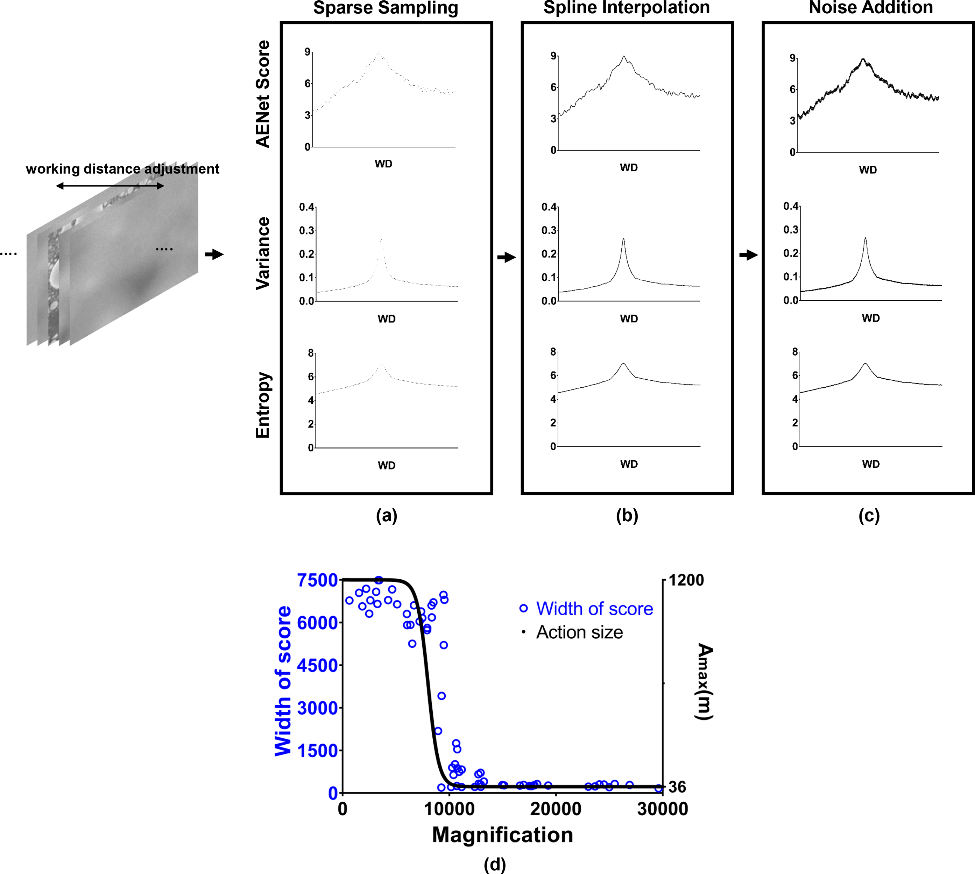


**Supplementary Figure. S1** **a-c** Process of constructing a dataset for training the ACNet and example data. **a** Autofocusing-evaluation network (AENet) score, variance, and entropy values calculated from the acquired images of Dataset 2 in the faster imaging mode at sparse working distance intervals. **b** Spline interpolation of the sparse samples. **c** Dataset prepared for ACNet training, which is the result of adding random noise to **b**. Range of random noise was empirically adjusted to match actual noise presented in the original data. **d** Plot of the width of the autofocusing-evaluation network (AENet) score distribution according to the working distance (left) and the determined maximum action size for the autofocusing-control network (ACNet; right). Based on the fact that the higher the magnification the narrower the width of the score, maximum action size was adaptively adjusted according to magnification with the fitted hyperbolic tangent function.

| **ACNet result** | **Tin ball** | **TEM grid** | **Gold nanoparticles** |
| --- | --- | --- | --- |
| **Trained with AENet score, conventional image quality metrics, and SEM parameters** | | | |
| Average of selected score | 8.468±1.015 | 8.605±0.899 | 7.788±1.380 |
| Average of score diff | 0.158±0.414 | 0.152±0.310 | 0.068±0.071 |
| Average of iteration | 9.100±4.154 | 9.100±4.128 | 9.947±4.075 |
| MSE | 0.020±0.008 | 0.021±0.012 | 0.001±0.000 |
| **Trained without the AENet score** | | | |
| Average of selected score | 8.111±1.177 | 8.051±1.478 | 7.328±1.401 |
| Average of score diff | 0.371±0.369 | 0.763±0.383 | 0.501±0.629 |
| Average of iteration | 15.200±3.665 | 14.800±3.778 | 12.947±2.915 |
| MSE | 0.062±0.033 | 0.043±0.058 | 0.021±0.019 |
| **Trained without the conventional image quality metrics** | | | |
| Average of selected score | 8.223±1.563 | 7.953±1.450 | 7.329±1.404 |
| Average of score diff | 0.280±0.678 | 0.862±0.283 | 0.457±0.617 |
| Average of iteration | 10.900±4.242 | 10.950±4.097 | 12.421±5.650 |
| MSE | 0.059±0.042 | 0.049±0.084 | 0.024±0.047 |

**Supplementary Table S1.** Comparison of offline test results of the proposed autofocusing-control network (ACNet) and its variation with different combination of input data to investigate whether both the autofocusing-evaluation network (AENet) score and the conventional image quality metrics can contribute to the improvement of the autofocus capability.

| **Layer** | | **Filter Size** | **Stride** | **Padding** | **Number of Filters** | **Normalization** | **Activation Function** | **Output Feature Map Size** |
| --- | --- | --- | --- | --- | --- | --- | --- | --- |
| # | type |  |  |  |  |  |  |  |
| 1 | Conv | 5ⅹ5ⅹ2 | 1 | 2 | 3 | Batch 2D | LeakyReLU | 320ⅹ240ⅹ2 |
| 2 | Conv | 4ⅹ3ⅹ3 | 1 | 0 | 32 | Batch 2D | LeakyReLU | 317ⅹ238ⅹ3 |
| 3 | Conv | 4ⅹ3ⅹ32 | 1 | 0 | 64 | Batch 2D | LeakyReLU | 314ⅹ236ⅹ32 |
| 4 | Conv | 4ⅹ3ⅹ64 | 1 | 0 | 64 | Batch 2D | LeakyReLU | 311ⅹ234ⅹ64 |
| 5 | Conv | 4ⅹ3ⅹ64 | 1 | 0 | 64 | Batch 2D | LeakyReLU | 308ⅹ232ⅹ64 |
|  | Conv  (Residual Connection) | 7ⅹ5ⅹ32 | 1 | 0 | 64 | Batch 2D | LeakyReLU | 308ⅹ232ⅹ64 |
| 6 | MaxPooling | 2ⅹ2 | 2 | 0 |  |  |  | 154ⅹ116ⅹ64 |
| 7 | Conv | 4ⅹ3ⅹ64 | 1 | 0 | 128 | Batch 2D | LeakyReLU | 151ⅹ114ⅹ128 |
| 8 | Conv | 4ⅹ3ⅹ128 | 1 | 0 | 128 | Batch 2D | LeakyReLU | 148ⅹ112ⅹ128 |
|  | Conv  (Residual Connection) | 7ⅹ5ⅹ64 | 1 | 0 | 128 | Batch 2D | LeakyReLU | 148ⅹ112ⅹ128 |
| 9 | MaxPooling | 2ⅹ2 | 2 | 0 |  |  |  | 74ⅹ56ⅹ128 |
| 10 | Conv | 4ⅹ3ⅹ128 | 1 | 0 | 256 | Batch 2D | LeakyReLU | 71ⅹ54ⅹ256 |
| 11 | Conv | 4ⅹ3ⅹ256 | 1 | 0 | 256 | Batch 2D | LeakyReLU | 68ⅹ52ⅹ256 |
|  | Conv  (Residual Connection) | 7ⅹ5ⅹ128 | 1 | 0 | 256 | Batch 2D | LeakyReLU | 68ⅹ52ⅹ256 |
| 12 | MaxPooling | 2ⅹ2 | 2 | 0 |  |  |  | 34ⅹ26ⅹ256 |
| 13 | Conv | 5ⅹ4ⅹ256 | 1 | 0 | 512 | Batch 2D | LeakyReLU | 30ⅹ23ⅹ512 |
| 14 | Conv | 5ⅹ4ⅹ512 | 1 | 0 | 512 | Batch 2D | LeakyReLU | 26ⅹ20ⅹ512 |
|  | Conv  (Residual Connection) | 9ⅹ7ⅹ256 | 1 | 0 | 512 | Batch 2D | LeakyReLU | 26ⅹ20ⅹ512 |
| 15 | MaxPooling | 2ⅹ2 | 2 | 0 |  |  |  | 13ⅹ10ⅹ512 |
| 16 | Conv | 5ⅹ4ⅹ512 | 1 | 0 | 512 | Batch 2D | LeakyReLU | 9ⅹ7ⅹ512 |
| 17 | Conv | 5ⅹ4ⅹ512 | 1 | 0 | 512 | Batch 2D | LeakyReLU | 5ⅹ4ⅹ512 |
|  | Conv  (Residual Connection) | 9ⅹ7ⅹ512 | 1 | 0 | 512 | Batch 2D | LeakyReLU | 5ⅹ4ⅹ512 |
| 18 | Conv | 5ⅹ4ⅹ512 | 1 | 0 | 512 | Batch 2D | LeakyReLU | 1ⅹ1ⅹ512 |
| 19 | Conv | 1ⅹ1ⅹ512 | 1 | 0 | 256 | Batch 2D | LeakyReLU | 1ⅹ1ⅹ256 |
| 20 | Conv | 1ⅹ1ⅹ256 | 1 | 0 | 128 | Batch 2D | LeakyReLU | 1ⅹ1ⅹ128 |
| 21 | Conv1 | 1ⅹ1ⅹ128 | 1 | 0 | 1 |  | Modified ReLU | 1ⅹ1ⅹ1 |

**Supplementary Table S2.** Architecture of the autofocusing-evaluation network (AENet).

| All elements of the state vector $\boldsymbol{(n}_{\boldsymbol{elements}}\boldsymbol{=80)}$ | | | |
| --- | --- | --- | --- |
|  | Current value | Differences | Normalization factor |
| Working distance terms  (n = 19) | ­­­- | $\boldsymbol{WD}_{\boldsymbol{t}}\text{ -}\boldsymbol{WD}_{\boldsymbol{t-i}}\boldsymbol{(i=1,2,3,\ldots,19)}$ | $\max\boldsymbol{action size}$ |
| Score terms  (n = 20) | $\boldsymbol{score}_{\boldsymbol{t}}$ | $\boldsymbol{score}_{\boldsymbol{t}}\text{ -}\boldsymbol{score}_{\boldsymbol{t-i}}\boldsymbol{(i=1,2,3,\ldots,19)}$ | $\boldsymbol{10}$ |
| Variance terms  (n = 20) | $\boldsymbol{var}_{\boldsymbol{t}}$ | $\boldsymbol{var}_{\boldsymbol{t}}\text{ -}\boldsymbol{var}_{\boldsymbol{t-i}}\boldsymbol{(i=1,2,3,\ldots,19)}$ | - |
| Entropy terms  (n = 20) | $\boldsymbol{ent}_{\boldsymbol{t}}$ | $\boldsymbol{ent}_{\boldsymbol{t}}\text{ -}\boldsymbol{ent}_{\boldsymbol{t-i}}\boldsymbol{(i=1,2,3,\ldots,19)}$ | $\boldsymbol{10}$ |
| Magnification  (n = 1) | $\boldsymbol{mag}_{\boldsymbol{t}}$ | - | $\boldsymbol{10}^{\boldsymbol{5}}$ |

Supplementary Table S3. The input of the autofocusing-control network (ACNet)

**Supplementary Movie S1. Autofocusing SEM demonstration movie for tin balls at X500.** Supplementary movie 1, which shows the autofocusing SEM demo frame-by-frame, is available online. The left figure shows the scores based on the adjustment of the WD in the pre-acquired WD-score graph, and the right image shows the corresponding image.

**Supplementary Movie S2. Autofocusing SEM demonstration movie for tin balls at X2,968.**

**Supplementary Movie S3. Autofocusing SEM demonstration movie for TEM grids at X500.**

**Supplementary Movie S4. Autofocusing SEM demonstration movie for TEM grids at X5,000.**

**Supplementary Movie S5. Autofocusing SEM demonstration movie for gold nanoparticles at X20,000.**

**Supplementary Movie S6. Autofocusing SEM demonstration movie for gold nanoparticles at X10,000.**

**Supplementary Movie S7. Autofocusing SEM demonstration movie for butterfly wing at X1000.**

**Supplementary Movie S8. Autofocusing SEM demonstration movie for piece of fabric at X200.**
